# Investigating psychometric properties of the Thai version of the Zarit Burden Interview using rasch model and confirmatory factor analysis

**DOI:** 10.1186/s13104-020-04967-w

**Published:** 2020-03-02

**Authors:** Kanokporn Pinyopornpanish, Manee Pinyopornpanish, Nahathai Wongpakaran, Tinakon Wongpakaran, Atiwat Soontornpun, Pimolpun Kuntawong

**Affiliations:** 1 Department of Family Medicine, Faculty of Medicine, Chiang Mai University, Chiang Mai, Thailand; 2 Geriatric Psychiatry Unit, Department of Psychiatry, Faculty of Medicine, Chiang Mai University, 110 Intawaroros Rd., T. Sriphum, A. Muang, Chiang Mai, Thailand; 3 Division of Neurology, Department of Internal Medicine, Faculty of Medicine, Chiang Mai University, Chiang Mai, Thailand

**Keywords:** Zarit Burden Interview (ZBI), Alzheimer’s disease, Caregivers, Rasch analysis, Perceived stress, Depression, Psychometric property

## Abstract

**Objective:**

The Zarit Burden Interview (ZBI) has been widely used to assess caregiver burden. Few research papers have investigated the Thai version of the ZBI. The study aimed to examine the psychometric properties of the Thai version of both the full length (ZBI-22) and short versions (ZBI-12) using Rasch analysis and confirmatory factor analysis among a sample of Alzheimer’s disease caregivers.

**Results:**

The ZBI-22 fitted the Rasch measurement model regarding unidimensionality but not for ZBI-12. Five items from ZBI-22, and 2 items from ZBI-12 were shown to be misfitting items. Half of ZBI items were shown to be disordered category or threshold, and were locally dependent. CFA revealed three-factor and four-factor fitted the data the best for ZBI-22 and ZBI-12, respectively. Reliability was good for both forms of the ZBI (α = 0.86–0.92). Significant correlations were found with caregiver’s perceived stress, anxiety/depression, pain and mobility but not with self-care and usual activity (*p* > 0.05), indicating convergent and discriminant validity. To conclude, the Thai version ZBI-22, but not ZBI-12, supported the reliability and unidimensional scale among Alzheimer’s disease caregivers. Some misfitting items of the ZBI undermined the unidimensionality of the scale, and need revision.

## Introduction

Caregiver burden is a state where the physical and psychological well-being, family relations or financial status of the caregiver could be threatened by providing the necessary care to another [1]. Caregiving, especially for elderly with dementia, usually causes burden among caregivers [2–5]. Studies showed that burden can ultimately lead to depression [6], and could lead to the poor treatment outcomes of the patients [7].

Thailand is becoming an aging society which would increase the number of dependent individuals and tendency of a household’s need for caregivers [8], so the burden of caregivers should be concerned. Studies have shown that over 40 to 70% of caregivers perceiving a burden [9, 10].

One of the oldest and most common measurements to assess dementia caregiver burden is the Zarit Burden Interview (ZBI) [11, 12]. It measures multidimensional aspects including physical, emotional, financial and social burden and the relation with the care receiver. It originated as a 29-item questionnaire but currently has been translated to many languages and revised to a 22-item form (ZBI-22) [13]. Shorter forms of ZBI have been developed by researchers over the past decades, ranging from 1 to 14 items [14]. However, the most widely used is ZBI-12, introduced by Be´dard et al. [15]. ZBI-12 has shown good psychometric properties in various languages and cultures [16–25]. Regarding its factor structure, the dimensions of ZBI range from 2 to 5 [15, 20]. Due to its multidimensional nature, it may not be accurately captured by a global score [26–28]. The ZBI has demonstrated high correlations with other psychological tools [17, 23–25, 29]. For ZBI-12, factor analysis revealed a two-factor rather than a unidimensional model despite being shorter [27]. Correlations between the ZBI-12 and ZBI-22 received a value of 0.96 in the initial study. To capture the global score of burden, the unidimensional ZBI was developed using item response theory (IRT), yielding a different set of items for the short scale as compared with the former 12-item ZBI [30].

To our knowledge, only one study examined the Thai version of ZBI-12 using factor analysis [31]. The Thai ZBI has never been tested for psychometric properties among Thai dementia caregivers and using IRT or Rasch measurement model. Therefore, the present study aimed to examine the ZBI construct by means of convergent, discriminant and concurrent validity, using both Rasch analysis and confirmatory factor analysis (CFA).

## Main text

### Methods

#### Subjects

One hundred and two caregivers of patients with Alzheimer’s, who were diagnosed and treated by neurologists at Maharaj Nakorn Chiang Mai Hospital, participated in the study. Primary caregivers aged 18 years old or more, who had been providing care for at least 1 month were recruited. Exclusion criteria was inability to communicate due to either language barrier or severe mental health problem.

#### Data collection

Data were collected at an outpatient clinic through structured interviews by one physician (MP) who had no role in patient care planning. All gave written informed consent before completing the questionnaires. The questionnaires included sociodemographic data, records related to caregiving and specific measures, which were ZBI, Perceived stress scale (PSS), Patient Health Questionnaire (PHQ-9), and EQ-5D.

#### Outcome measures

##### ZBI

The ZBI is a caregiver-reported questionnaire measuring the burden the respondent feels in providing care to the patient. Currently, it has two widely used forms, ZBI-22 and ZBI-12, with a Likert scoring scale between 0 (never) and 4 (nearly always) [15, 32]. Studies showed high correlation in both ZBI-22 and ZBI-12 with the Caregiver Activity Survey, and with other tools [25, 33].

The Thai version (translated version) of the ZBI used in this study was allowed by Professor Zarit and Mapi Research Trust [13]. The study sample showed a Cronbach’s alpha of 0.921 for the ZBI-22 and 0.865 for ZBI-12.

##### PSS

The PSS is a self-reporting, 10-item questionnaire measuring the extent to which individuals perceived stress [34]. The 4-response Likert scale, ranges from 0 (not at all) to 4 (the most). The Thai version PSS showed a Cronbach’s alpha of 0.85. It correlated with other measures including the State Trait Anxiety Inventory, but negatively correlated with the Rosenberg Self-Esteem Scale [35]. The study sample showed a Cronbach’s alpha of 0.850.

##### PHQ-9

The PHQ-9 is a self-reporting, 9-item questionnaire measuring the extent to which an individual feels bothered due to depressive symptoms over the past 2 weeks [36]. The 4-response Likert scale ranges from 0 (not at all) to 3 (nearly every day). The Thai version PHQ-9 showed a Cronbach’s alpha of 0.79 and a positive association between the PHQ-9 and the HAM-D [37]. The study sample showed a Cronbach’s alpha of 0.849.

##### EQ-5D

The EQ-5D is a self-reporting questionnaire measuring health-related quality of life [38]. It comprises 5 items assessing 5 domains of health state: mobility, self-care, usual activities, pain and anxiety/depression, with a 5-response scale ranging from 1 (no problem) to 5 (severe problem). All 5 aspects were calculated to an index score with the maximum of 1.000 [39]. An intraclass correlation coefficient of 0.987 for the EQ-5D index score, and a significant correlation with WHOQOL-BREF were noted [40]. The study sample showed that Cronbach’s alpha was 0.723.

#### Statistical analysis

Sociodemographic data were analyzed using descriptive statistics. Pearson’s or Spearman’s rank was used for correlational analysis. The same items were presented in both tests, leading to an overestimate of the “true” correlation, so a corrected correlation was made between both forms of ZBI [41].

Based on measurement theory, a scale should demonstrate that all items contribute to the same construct, and has monotonically increasing steps. All these properties can be illustrated by the Rasch model. The following approach was conducted for analysis.

##### Correlation analysis

We tested the ZBI against the EQ5D subscale, hypothesizing that ZBI should relate more to anxiety/depression than mobility. We expected to find a low—moderate correlation between ZBI and PSS and PHQ-9 to demonstrate concurrent validity.

##### Rasch analysis

The Rasch model belongs to the item-response latent trait models, a probabilistic logistic model that predicts that the response to a particular item is influenced by the quality of both person and item. More details can be found elsewhere [42]. The partial credit Rasch model was used [43], with the following criteria. First, unidimensionality and local independence, which were evaluated by (a) the first principal component of the residuals (or first contrast) should have an eigen value less than 2, (b) disattenuated correlation > 0.7 and (c) item fit statistics (INFIT and OUTFIT mean-square) indicating the consistency of each item to the other items, should be 0.70 and 1.30 [44]. To evaluate local independency, a standardized residual correlation should be less than 0.3 [45]. Second, response category functioning and ordered categories and thresholds are expected for measurement [46]. Third, a reliability coefficient of 0.80 or higher and of 0.90 or higher are considered acceptable for person reliability and item reliability, respectively.

##### CFA

To test how data were well modeled with the unidimensional construct, CFA was performed for both ZBI-22 and ZBI-12. The Weighted Least Square Mean and Variance corrected method of estimation was used for the nonnormality and ordinal types of items. Assessment model fit used Chi square (p > 0.05), comparative fit index and Tucker Lewis Index, where values 0.95 or higher are preferable [47]. Root mean square error of approximation value < 0.08 was indicative of an acceptable model fit [48].

##### Computer software

For CFA, Mplus, Version 8.4 was used (Muthén and Muthén 2015). Rasch analysis used Winsteps, Version 4.4.8 (Beaverton, Oregon: Winsteps.com). All other analyses were performed using IBM SPSS, Version 22 (SPSS Inc., Chicago, IL, USA).

## Results

The average age of the caregiver sample was 55 years (SD = 12.9); most were women (77.5%). According to ZBI level, the sample was reported to have low burden. The quality of life index score was quite high on average, while perceived stress and depressive symptoms were low (Table 1).

**Table 1:** Demographic characteristics of participants

Demographic characteristics	Number (%) or mean (SD)
Age (year)–mean (SD)	55.0 (12.9)
Sex, female	79 (77.5)
Years of education	13.8 (4.6)
Relationship to the patient	
Spouse	21 (20.6)
Parents	1 (1.0)
Offspring	70 (68.6)
Relatives	5 (4.9)
Nonrelated (hired caregiver)	5 (4.9)
Length of caregiving, median, IQR, min–max (years)	3, 5, (0.3–50.0)
Number of hours per day	15.4 (8.1)
Number of days per week	6.3 (1.4)
Having physical illness	53 (52.0)
Having mental problems	8 (7.8)
Clinical data	
ZBI-22	18.4 (14.3)
ZBI-12	10.6 (7.49)
EQ-5D	0.88 (0.15)
Mobility	1.47 (0.83)
Self-care	1.17 (0.69)
Usual activities	1.32 (0.77)
Pain/discomfort	1.90 (0.79)
Anxiety/depression	1.63 (0.80)
PSS	14.6 (4.95)
PHQ-9	4.11 (3.96)

SD, standard deviation; IQR, interquartile range; ZBI, Zarit Burden Interview; PHQ-9, Patient Health Questionnaire-9; PSS, Perceived stress scale

For the distribution of the ZBI-items, some had unacceptable kurtosis (> ±3), which contributed to the high frequency of zero categories on these respective items (Additional file 1: Table S1).

Correlation analysis showed that ZBI-22 had a coefficient of 0.855 (p < 0.01) with ZBI-12 for the uncorrected correlation, and 0.784 (p < 0.01). Both ZBI-22 and ZBI-21 significantly related to PHQ-9, PSS, the EQ-5D index score, subscale mobility, pain and anxiety/depression, but not to self-care and usual activity indicating convergent and discriminant validity (Table 2).

**Table 2:** Zero correlation between variables

	1	2	3	4	5	6	7	8	9	10
1. ZBI-12	1	0.855^**^	0.281^**^	0.001	0.153	0.377^**^	0.466^**^	− 0.366^**^	0.540^**^	0.452^**^
2. ZBI-22		1	0.289^**^	0.036	0.120	0.364^**^	0.418^**^	− 0.356^**^	0.510^**^	0.366^**^
3. EQ-5D: mobility			1	0.341^**^	0.481^**^	0.451^**^	0.240^*^	− 0.738^**^	0.356^**^	0.122
4. EQ-5D: self-care				1	0.534^**^	0.127	0.118	− 0.644^**^	0.273^**^	0.109
5. EQ-5D: usual activities					1	0.291^**^	0.380^**^	− 0.778^**^	0.473^**^	0.223^*^
6. EQ-5D: pain/discomfort						1	0.488^**^	− 0.623^**^	0.426^**^	0.166
7. EQ-5D: anxiety/depression							1	− 0.617^**^	0.590^**^	0.379^**^
8. EQ-5D Utility index								1	− 0.623^**^	− 0.298^**^
9. PHQ-9									1	0.392^**^
10. PSS										1

ZBI, Zarit Burden Interview; PHQ-9, Patient Health Questionnaire-9; PSS, Perceived stress scale

*p < 0.05, **p < 0.01

Rasch analysis results showed that the unexplained variance in the first contrast yielded eigen values of 2.52 and 3.03, implying a possible second dimension. However, based on disattenuated correlation between person measure > 0.7, the second dimension could noise for ZBI-22. Five items of ZBI-22, and two items of ZBI-12 were shown to be misfitted. Five pairs of items from ZBI-22 and three pairs of items from ZBI-12 had standardized residual correlations above 0.2, indicating item dependency of both forms of ZBI. For category function, 33 to 50% of items were found to be disordered category or threshold (Table 3). For this reason, the four original rating categories were combined in different ways until the criteria were best met. This was obtained by rescaling as follows: 0 = 0; 1 = 1; 2 = 2; 3 = 3 and 4 = 3 for ZBI-22, and 0 = 0; 1 = 1; 2 = 2; 3 = 2 and 4 = 3 for ZBI-12. After rescaling, the data fit better with Rasch model as the misfitting items reduced while reliability increased. All reliability values were shown to be in an acceptable range.

**Table 3:** Rasch analysis results of the ZBI

ZBI-22					ZBI-12				
No.	Measure	INFIT MNSQ	OUTFIT MNSQ	Disordered category or threshold	No.	Measure	INFIT MNSQ	OUTFIT MNSQ	Disordered category or threshold
1 Asks for more help than needs	0.44	1.321	1.453	Yes					No
2 Not have enough time	0.04	0.912	0.859	No	1	− 0.19	0.903	0.897	No
3 Feel stressed	− 0.03	0.697	0.614	Yes	2	− 0.65	0.905	0.865	No
4 Feel embarrassed	0.14	1.120	1.099	Yes					
5 Feel angry	0.13	0.891	0.932	No	3	0.40	0.978	1.014	No
6 Negative relationships	0.37	0.941	0.956	Yes	4	0.67	1.129	0.855	Yes
7 Afraid about the future	− 0.64	1.082	1.134	No					
8 Dependent on you	− 0.92	1.258	1.334	Yes					
9 Feel strained	0.23	0.760	0.655	No	5	0.33	0.818	0.781	Yes
10 Health decreased	0.31	0.646	0.795	No	6	0.40	0.806	1.083	No
11 Lack of privacy	− 0.04	0.616	0.730	No	7	0.11	0.893	1.062	No
12 Lack of social life	0.19	0.675	0.639	No	8	0.32	0.701	0.682	No
13 Feel uncomfortable	0.38	0.943	1.967	No					
14 Expecting to be cared for	− 0.58	1.512	1.612	Yes					
15 Lack of money	0.12	1.355	1.399	No					
16 Unable to care much longer	0.72	0.789	0.777	Yes					
17 Lost control of life	0.60	0.844	0.737	Yes	9	0.35	0.918	0.796	Yes
18 Leave the care	0.40	1.423	1.358	Yes					
19 Uncertain about what to do	0.14	0.795	0.704	Yes	10	0.40	1.035	1.029	Yes
20 Should do more	− 0.71	1.264	1.256	No	11	− 1.08	1.406	1.339	No
21 Could do a better job caring	− 0.80	1.383	1.367	No	12	− 1.06	1.449	1.317	No
22 Overall feeling of burden	− 0.50	0.912	0.973	Yes					

PCA	ZBI-22	ZBI-12
%Variance explained by measures	46.0%	48.7%
Unexplained variance in first contrast	2.52 (6.2%)	3.03 (12.6%)
Disattenuated correlation	0.896–1.000	0.357–1.000
Pair of item that standardized residual correlation > 0.2	#20(should do more)—#21(should do more) #11(lack of privacy)—#12(lack of social life) #1(asks for more help than needs)—#18(leave the care) #15(lack of money)—#16(unable to care much longer) #5(feel angry)—#9(feel strained)	#11(should do more)—#12(should do more) #3(feel angry)—#5(feel strained) #8(lack of privacy)—#9(lack of social life)
Item separation/reliability	2.57/0.87	2.03/0.80
Person separation/reliability	4.08/0.94	3.97/0.94

ZBI, Zarit Burden Interview; PCA, Principal component analysis; INFIT, inlier-pattern-sensitive fit statistic; OUTFIT, outlier-sensitive fit statistic; MNSQ, mean-square; #, item number

The CFA showed that the unidimensional model did not fit with the data for both versions of ZBI. Three-factor model provided the best-fitted statistics for ZBI-22, while the four-factor model with the correlated error terms of items 11 and item 12, provided the best-fitted statistics for ZBI-12 (Additional file 2: Table S2).

## Discussion

The present study aimed to evaluate the psychometric properties of the Thai version of the ZBI among caregivers of patients with Alzheimer’s disease. Consistent with related studies, both ZBI-22 and ZBI-12 did not demonstrate a unidimensional scale [49, 50], even though the ZBI-22 seemed to be favored over ZBI-12. Three-factor and four-factor fitted the data the best for ZBI-22 and ZBI-12, respectively. However, the disattenuated correlation (> 0.70) in ZBI-22 suggested that it could be sufficiently unidimensional, but not for ZBI-12.

Pairs of error variances to be correlated suggested by CFA corresponded to local dependence by Rasch analysis. This was consistent with Ballesteros et al.’s study [30] in that both items, “should do more” and “could do a better job caring” were excluded from the new 12-item ZBI. In addition to these two items, more pairs were shown to be locally dependent. Violations of local independence in a unidimensional scale can lead to inflated estimates of reliability, providing a false impression of the accuracy and precision of estimates [51].

Disordered categories and thresholds indicated that respondents had difficulty discriminating between response categories given their level of caregiver burden. In ZBI-22, the response categories were collapsed from five to four categories and by that category 3 (quite frequently) and 4 (nearly always) were collapsed together. Oddly, for ZBI-12, collapsing category 2 (sometimes) and 3 (quite frequently) yielded better results. It remains unclear why the participants responded differently to the same items of different scales.

Suggestions from our findings are twofold if interpretation of mean scores, or changes in total scores is to be meaningful, First, is to revise or remove the locally dependent and misfitting items 11 and 12 of ZBI-12 to make it better unidimensional scale. Second, is to look for the fitted items with ordered category and threshold from ZBI-22 to form a new short ZBI.

Taken together, the Thai version of ZBI-12 may not be regarded as unidimensional, an interval rating scale of burden among caregivers to patients with Alzheimer’s disease. The ZBI-22 showed sufficient unidimensionality. Some items were suggested to be removed if ZBI-12 is to be used.

## Limitations and future study

Clinicians should interpret the results in light of the limitation in sample size. Replication in a larger sample size should be encouraged. Test–retest reliability, sensitivity to change and equivalence test in different populations and cultures should be warranted.

## Supplementary information

**Additional file 1: Table S1.** Descriptive data of the full-length and the short ZBI.

**Additional file 2: Table S2.** Fit indices of various proposed models of the ZBI.

## Data Availability

The datasets used and/or analyzed during the current study are available from the corresponding author on reasonable request.

## Abbreviations

ZBI: Zarit Burden Interview
CFA: Confirmatory factor analysis
PCA: Principal component analysis
RMSEA: Root mean square error of approximation
CFI: Comparative fit index
TLI: Tucker lewis index
WLSMV: Weighted Least Square Mean and Variance Corrected
