# Correction to: Investigating psychometric properties of the Thai version of the Zarit Burden Interview using rasch model and confirmatory factor analysis

**DOI:** 10.1186/s13104-020-05051-z

**Published:** 2020-04-20

**Authors:** Kanokporn Pinyopornpanish, Manee Pinyopornpanish, Nahathai Wongpakaran, Tinakon Wongpakaran, Atiwat Soontornpun, Pimolpun Kuntawong

**Affiliations:** 1 Department of Family Medicine, Faculty of Medicine, Chiang Mai University, Chiang Mai, Thailand; 2 Department of Psychiatry, Faculty of Medicine, Chiang Mai University, 110 Intawaroros Rd. Tambon Sriphoom, Amphur Mueng, Chiang Mai, 50200 Thailand; 3 Division of Neurology, Department of Internal Medicine, Faculty of Medicine, Chiang Mai University, Chiang Mai, Thailand

## Correction to: BMC Res Notes (2020) 13:120 10.1186/s13104-020-04967-w

Following the publication of the original article [1], the authors unfortunately became aware of the following errors in Table 3:

#21 in ZBI-22 should be corrected from ‘should do more’ to ‘could do a better job’.

#12 in ZBI-12 should be corrected from ‘should do more’ to ‘could do a better job’.

The corrected Table 3 is shown below:

**Table 3:** Rasch analysis results of the ZBI

ZBI-22					ZBI-12				
No.	Measure	INFIT MNSQ	OUTFIT MNSQ	Disordered category or threshold	No.	Measure	INFIT MNSQ	OUTFIT MNSQ	Disordered category or threshold
1. Asks for more help than needs	0.44	1.321	1.453	Yes					No
2. Not have enough time	0.04	0.912	0.859	No	1	− 0.19	0.903	0.897	No
3. Feel stressed	− 0.03	0.697	0.614	Yes	2	− 0.65	0.905	0.865	No
4. Feel embarrassed	0.14	1.120	1.099	Yes					
5. Feel angry	0.13	0.891	0.932	No	3	0.40	0.978	1.014	No
6. Negative relationships	0.37	0.941	0.956	Yes	4	0.67	1.129	0.855	Yes
7. Afraid about the future	− 0.64	1.082	1.134	No					
8. Dependent on you	− 0.92	1.258	1.334	Yes					
9. Feel strained	0.23	0.760	0.655	No	5	0.33	0.818	0.781	Yes
10. Health decreased	0.31	0.646	0.795	No	6	0.40	0.806	1.083	No
11. Lack of privacy	− 0.04	0.616	0.730	No	7	0.11	0.893	1.062	No
12. Lack of social life	0.19	0.675	0.639	No	8	0.32	0.701	0.682	No
13. Feel uncomfortable	0.38	0.943	1.967	No					
14. Expecting to be cared for	− 0.58	1.512	1.612	Yes					
15. Lack of money	0.12	1.355	1.399	No					
16. Unable to care much longer	0.72	0.789	0.777	Yes					
17. Lost control of life	0.60	0.844	0.737	Yes	9	0.35	0.918	0.796	Yes
18. Leave the care	0.40	1.423	1.358	Yes					
19. Uncertain about what to do	0.14	0.795	0.704	Yes	10	0.40	1.035	1.029	Yes
20. Should do more	− 0.71	1.264	1.256	No	11	− 1.08	1.406	1.339	No
21. Could do a better job caring	− 0.80	1.383	1.367	No	12	− 1.06	1.449	1.317	No
22. Overall feeling of burden	− 0.50	0.912	0.973	Yes					

PCA	ZBI-22	ZBI-12
%Variance explained by measures	46.0%	48.7%
Unexplained variance in first contrast	2.52 (6.2%)	3.03 (12.6%)
Disattenuated correlation	0.896–1.000	0.357–1.000
Pair of item that standardized residual correlation > 0.2	#20(should do more)—#21(could do a better job) #11(lack of privacy)—#12(lack of social life) #1(asks for more help than needs)—#18(leave the care) #15(lack of money)—#16(unable to care much longer) #5(feel angry)—#9(feel strained)	#11(should do more)—#12(could do a better job) #3(feel angry)—#5(feel strained) #8(lack of privacy)—#9(lack of social life)
Item separation/reliability	2.57/0.87	2.03/0.80
Person separation/reliability	4.08/0.94	3.97/0.94

ZBI, Zarit Burden Interview; PCA, Principal component analysis; INFIT, inlier-pattern-sensitive fit statistic; OUTFIT, outlier-sensitive fit statistic; MNSQ, mean-square; #, item number

## References

[1] Pinyopornpanish K, Pinyopornpanish M, Wongpakaran N, Wongpakaran T, Soontornpun A, Kuntawong P (2020). Investigating psychometric properties of the Thai version of the Zarit Burden Interview using rasch model and confirmatory factor analysis. BMC Res Notes.

