# Supplementary material for: Investigating psychometric properties of the Thai version of the Zarit Burden Interview using rasch model and confirmatory factor analysis
Source: BMC Res Notes. 2020 Mar 2;13:120. doi: 10.1186/s13104-020-04967-w (PMC7050141; doi:10.1186/s13104-020-04967-w)
Supplement: Supplementary file 1 — Additional file 1: Table S1. Descriptive data of the full-length and the short ZBI. [file 13104_2020_4967_MOESM1_ESM.docx]

| **Table S1** Descriptive data of the full-length and the short ZBI | | | | | | | | | | | | | | | |
| --- | --- | --- | --- | --- | --- | --- | --- | --- | --- | --- | --- | --- | --- | --- | --- |
|  | **ZBI-22** | | | | | | | **ZBI-12** | | | | | | | |
| **No.** | Mean | Median | S.D. | Skew | Kurtosis | Min | Max | No. | Mean | Median | S.D. | Skew | Kurtosis | Min | Max |
|  | 18.44 | 15.00 | 14.27 | 0.90 | 0.46 | 0.00 | 66.00 |  | 10.65 | 10.00 | 7.50 | 1.25 | 2.62 | 0 | 39 |
| 1 asks for more help than needs | 0.60 | 0.00 | 0.91 | 1.53 | 1.87 | 0 | 4 | - | - | - | - | - | - | - | - |
| 2 not have enough time | 0.84 | 1.00 | 0.99 | 1.15 | 0.91 | 0 | 4 | 1 | 1.11 | 1.00 | 0.96 | 0.54 | 0.01 | 0 | 4 |
| 3 feel stressed | 0.95 | 1.00 | 1.05 | 0.89 | 0.00 | 0 | 4 | 2 | 1.34 | 1.00 | 1.10 | 0.59 | -0.33 | 0 | 4 |
| 4 feel embarrassed | 0.96 | 1.00 | 1.07 | 0.79 | -0.47 | 0 | 4 | - | - | - | - | - | - | - | - |
| 5 feel angry | 0.63 | 0.00 | 0.85 | 1.09 | 0.16 | 0 | 3 | 3 | 0.73 | 1.00 | 0.87 | 1.11 | 1.03 | 0 | 4 |
| 6 negative relationships | 0.54 | 0.00 | 0.81 | 1.25 | 0.47 | 0 | 3 | 4 | 0.46 | 0.00 | 0.81 | 2.01 | 4.24 | 0 | 4 |
| 7 afraid about the future | 1.28 | 1.00 | 1.18 | 0.68 | -0.31 | 0 | 4 | - | - | - | - | - | - | - | - |
| 8 dependent on you | 1.50 | 1.00 | 1.47 | 0.49 | -1.16 | 0 | 4 | - | - | - | - | - | - | - | - |
| 9 feel strained | 0.82 | 1.00 | 0.96 | 0.98 | 0.26 | 0 | 4 | 5 | 0.77 | 1.00 | 0.85 | 1.16 | 1.56 | 0 | 4 |
| 10 health decreased | 0.53 | 0.00 | 0.91 | 1.87 | 3.35 | 0 | 4 | 6 | 0.50 | 0.00 | 0.89 | 1.94 | 3.72 | 0 | 4 |
| 11 lack of privacy | 0.92 | 1.00 | 0.98 | 1.08 | 0.87 | 0 | 4 | 7 | 0.78 | 1.00 | 0.96 | 1.30 | 1.48 | 0 | 4 |
| 12 lack of social life | 0.68 | 0.00 | 0.93 | 1.45 | 2.01 | 0 | 4 | 8 | 0.54 | 0.00 | 0.94 | 1.91 | 3.25 | 0 | 4 |
| 13 feel uncomfortable | 0.48 | 0.00 | 0.92 | 2.02 | 3.63 | 0 | 4 | - | - | - | - | - | - | - | - |
| 14 expecting to be cared for | 1.14 | 1.00 | 1.39 | 0.95 | -0.46 | 0 | 4 | - | - | - | - | - | - | - | - |
| 15 lack of money | 0.61 | 0.00 | 1.03 | 1.85 | 2.75 | 0 | 4 | - | - | - | - | - | - | - | - |
| 16 unable to care much longer | 0.32 | 0.00 | 0.71 | 2.59 | 7.66 | 0 | 4 | - | - | - | - | - | - | - | - |
| 17 lost control of life | 0.44 | 0.00 | 0.79 | 2.00 | 4.14 | 0 | 4 | 9 | 0.55 | 0.00 | 0.87 | 1.95 | 4.30 | 0 | 4 |
| 18 leave the care | 0.45 | 0.00 | 0.84 | 2.17 | 5.09 | 0 | 4 | - | - | - | - | - | - | - | - |
| 19 uncertain about what to do | 0.73 | 0.00 | 0.96 | 1.33 | 1.47 | 0 | 4 | 10 | 0.69 | 0.00 | 0.90 | 1.32 | 1.40 | 0 | 4 |
| 20 should do more | 1.48 | 1.00 | 1.21 | 0.30 | -0.92 | 0 | 4 | 11 | 1.59 | 1.00 | 1.26 | 0.41 | -0.80 | 0 | 4 |
| 21 could do a better job caring | 1.46 | 1.00 | 1.27 | 0.43 | -0.83 | 0 | 4 | 12 | 1.57 | 2.00 | 1.28 | 0.41 | -0.78 | 0 | 4 |
| 22 Overall feeling of burden | 1.23 | 1.00 | 1.08 | 0.66 | -0.06 | 0 | 4 | - | - | - | - | - | - | - | - |
| ZBI = Zarit Burden Interview, S.D. = standard deviation | | | | | | | | | | | | | | | |
