# Supplementary material for: Investigating psychometric properties of the Thai version of the Zarit Burden Interview using rasch model and confirmatory factor analysis
Source: BMC Res Notes. 2020 Mar 2;13:120. doi: 10.1186/s13104-020-04967-w (PMC7050141; doi:10.1186/s13104-020-04967-w)
Supplement: Supplementary file 2 — Additional file 2: Table S2. Fit indices of various proposed models of the ZBI. [file 13104_2020_4967_MOESM2_ESM.docx]

| **Table S2.** Fit indices of various proposed models of the ZBI | | | | | | | | | | | | |
| --- | --- | --- | --- | --- | --- | --- | --- | --- | --- | --- | --- | --- |
| Model description | ZBI-22 | | | | | | ZBI-12 | | | | | |
|  | χ^2^ | df | p-value | RMSEA | CFI | TLI | χ^2^ | df | p-value | RMSEA | CFI | TLI |
| Single factor | 448.48 | 209 | .000 | 0.108 | 0.918 | 0.909 | 466.43 | 54 | .000 | 0.278 | 0.850 | 0.816 |
| Two-factor | 355.72 | 208 | .000 | 0.085 | 0.949 | 0.944 | 215.50 | 53 | .000 | 0.176 | 0.941 | 0.926 |
| Three-factor | 290.89 | 206 | .000 | 0.065 | 0.971 | 0.967 | 198.42 | 51 | .000 | 0.171 | 0.946 | 0.930 |
| Four-factor | 287.24 | 203 | .000 | 0.065 | 0.971 | 0.967 | 190.75 | 51 | .000 | 0.173 | 0.948 | 0.928 |
| Single factor* | - | - | - | - | - | - | 132.59 | 53 | .000 | 0.123 | 0.971 | 0.964 |
| Two-factor* | - | - | - | - | - | - | 101.99 | 52 | .000 | 0.099 | 0.982 | 0.977 |
| Three-factor* | - | - | - | - | - | - | 74.64 | 50 | .014 | 0.071 | 0.991 | 0.988 |
| Four-factor* | - | - | - | - | - | - | 65.01 | 47 | .042 | 0.062 | 0.993 | 0.991 |
| ZBI = Zarit burden interview, *= error term of item 11 and item 12 were correlated, df = degree of freedom, RMSEA = root mean square error of approximation , CFI= comparative fit index, TLI= Tucker- Lewis Index | | | | | | | | | | | | |
